# Prospective Ultrasonographic Evaluation of Adrenal Glands in a Population of Beagle Puppies and Functional Analysis of Basal Cortisol Levels in Blood

**DOI:** 10.3390/vetsci12050472

**Published:** 2025-05-14

**Authors:** Julia Topmöller, Kristina Merhof, Eva Packeiser, Marion Schmicke, Holger Andreas Volk, Johanna Rieder

**Affiliations:** 1 Department of Small Animal Medicine and Surgery, University of Veterinary Medicine Hannover, 30559 Hannover, Germany; 2 Veterinary-Endocrinology and Laboratory Diagnostics, Clinic for Cattle, University of Veterinary Medicine Hannover, Foundation, 30173 Hannover, Germany

**Keywords:** adrenocortical hypoadrenocorticism, cortisol, sonography, endocrinopathy

## Abstract

This study presents a prospective evaluation of adrenal gland development in a cohort of Beagle puppies, utilizing sonographic imaging and functional assessment of the basal serum cortisol concentrations. The objective was to characterize the growth patterns and physiological maturation of the adrenal glands in dogs between six and twelve months of age. Ten Beagle puppies from two litters, bred and maintained under standardized conditions at the Veterinary Teaching Hospital in Hannover, were included in the study. Serum cortisol levels were determined, and adrenal gland dimensions were measured using ultrasonography at regular intervals over a six-month observation period. The results demonstrated a significant growth trajectory of the adrenal glands, characterized by notable increases in both the longitudinal length and caudal pole diameter. No significant correlation was found between serum cortisol levels and adrenal gland size, indicating that the cortisol data primarily served to confirm the physiological function of the adrenal glands rather than to influence their size. The adrenal gland sizes mostly resembled those of adult animals; however, significant growth was observed between 6 and 12 months. In conclusion, the presence of small adrenal glands in young dogs with clinical suspicion should prompt further diagnostic testing or, at least, regular follow-up evaluations.

## Simple Summary

This study investigates adrenal gland development in ten Beagle puppies from two litters, raised under standardized conditions from 6 to 12 months of age. Adrenal gland size was assessed over a six-month period using ultrasonography, alongside the measurement of basal cortisol concentrations. A significant increase in adrenal gland size was observed during the study period; however, no correlation was identified between adrenal dimensions and baseline cortisol levels. These findings enhance current knowledge of adrenal maturation and may contribute to improved diagnostic approaches for adrenal disorders, such as hypoadrenocorticism, in juvenile dogs.

## 1. Introduction

Hypoadrenocorticism is characterized by a deficiency of both mineralocorticoids and glucocorticoids, causing, in some cases, nonspecific clinical signs, such as general weakness or gastrointestinal disturbances. A recent study reported that 4% of dogs presenting with chronic gastrointestinal clinical signs are affected by hypoadrenocorticism [1]. Consequently, nowadays, routine measurement of basal cortisol is recommended in dogs exhibiting chronic gastrointestinal signs. Although hypoadrenocorticism can affect dogs under one year of age, the exact prevalence in this age group remains unknown.

Diagnosing hypoadrenocorticism in younger dogs presents additional challenges, as certain indirect indicators of hypocortisolism—such as lymphocytosis—may be physiological in puppies and young animals [2]. In adult dogs, clinical suspicion is often supported by laboratory findings, including lymphocytosis, a decreased sodium-to-potassium ratio (<27), azotemia, and the absence of a stress leukogram, in combination with gastrointestinal clinical signs and general weakness [3,4]. While hypoadrenocorticism is most commonly reported in dogs between five and seven years of age, rare cases have been documented in individuals as young as two months to one year [5,6].

Abdominal ultrasonography is routinely employed in the diagnostic evaluation of dogs with gastrointestinal clinical signs, allowing for concurrent assessment of both the gastrointestinal tract and adrenal glands. While normative values for adrenal gland size are well established in adult dogs, further research is needed to define reference dimensions in growing individuals. Sonographic assessment may aid in raising clinical suspicion of hypoadrenocorticism [7,8,9]. Although numerous studies have demonstrated a relationship between body size and adrenal gland dimensions, to the authors’ knowledge, no data currently exist on adrenal growth patterns or the correlation between adrenal size and cortisol concentrations in dogs during their first year of life [8,10,11,12,13,14].

Despite its relatively low prevalence in adult dogs [1], hypoadrenocorticism requires a fundamentally different therapeutic approach involving mineralocorticoid and glucocorticoid supplementation compared to the management of other gastrointestinal disorders. Early detection is, therefore, crucial to improve patient outcomes and prevent life-threatening adrenal crises [15]. The aim of the present study was to conduct a prospective, longitudinal evaluation of adrenal gland morphology and size using ultrasonography, in conjunction with serum cortisol measurements, in a cohort of ten Beagle puppies raised under standardized environmental conditions.

## 2. Materials and Methods

Ultrasonographic evaluation of the adrenal glands was conducted as part of a prospective longitudinal study in ten Beagle puppies. Serum cortisol concentrations were measured to assess adrenal function. All procedures were approved by the relevant regulatory authority (Approval No. 33.19-42502-04-22-00179).

### 2.1. Study Population

The study population comprised ten Beagle puppies from two litters, bred in-house at the Veterinary Teaching Hospital for Companion Animals, University of Veterinary Medicine Hannover (Stiftung, Tierärztliche Hochschule Hannover). The cohort included five males and five females. All animals were clinically healthy prior to and throughout the study period. Data collection was conducted between November 2022 and April 2023, during which the puppies were between 6 and 12 months of age.

### 2.2. Blood Samples and Serum Cortisol Levels

All blood sampling and ultrasonographic imaging procedures were carried out in a standardized, veterinary-approved manner. Prior to the initial sampling, the dogs were acclimated to both the testing environment and the personnel involved in the procedures. To minimize stress and discomfort, the sampling process was paired with positive reinforcement techniques, and blood collection was performed prior to ultrasonographic examination [16,17].

Samples were collected at 6, 7, 8, 10, 11, and 12 months of age, at four-week intervals. For each time point, 3 mL of blood was drawn. Samples were allowed to clot at room temperature for 30 min before being centrifuged, and the resulting serum was stored at –80 °C until analysis. All samples were analyzed in a single batch at the conclusion of the study by the Clinical-Endocrinology Laboratory at the University of Veterinary Medicine Hannover using the chemiluminescence immunoassay (CLIA; Immulite 2000, Siemens Diagnostics, CA, USA) method [18]. The reference values ranged from 41.4 to 96.6 nmol/L, as validated by the Endocrinology Laboratory.

### 2.3. Sonographic Measurements of the Adrenal Gland

Ultrasonographic examinations were performed by a board-certified member of the European College of Veterinary Diagnostic Imaging. The imaging was conducted using an RS85 Prestige system (Samsung Medison, Seoul, Republic of Korea). Dogs were positioned in right lateral recumbency on an indented examination mat, and data were recorded immediately following each measurement. A microconvex or linear-array transducer was used, with the transducer frequency selected based on the individual animal’s body size and condition score, ranging from 7.5 to 18 MHz [19].

Adrenal gland architecture and echogenicity were assessed qualitatively. Quantitative parameters included the longitudinal length, measured in a sagittal plane, optimized to include the entire adrenal gland, the diameter of the caudal pole of both adrenal glands, and the diameter of the cranial pole of the left adrenal in transverse plane. The cranial pole of the right adrenal gland was excluded from transverse measurements due to its conical shape and location, which rendered it difficult to measure consistently and to avoid discomfort to the patient. Each measurement was repeated three times, and mean values were calculated. Mean growth rates were determined based on the changes observed between six months of age and the adult stage (10 to 12 months).

### 2.4. Statistics

Statistical analyses were conducted using GraphPad Prism 9 (GraphPad Software, Boston, MA, USA). Normal distribution was confirmed for the datasets corresponding to the longitudinal length of the left and right adrenal glands, as well as serum cortisol concentrations, using the Shapiro–Wilk test. These variables were subsequently analyzed using a one-way repeated-measures ANOVA with Tukey’s multiple-comparisons post hoc test. As the diameters of the left and right caudal poles and the left cranial pole did not follow a normal or log-normal distribution, the Friedman test with Dunn’s multiple-comparisons post hoc test was applied. For consistency, all data are presented as box-and-whisker plots. Males and females were compared at each time point and for each parameter using a *t*-test or Mann Whitney test, depending on the data distribution. The levels of significances were set to *p* < 0.05, *p* < 0.01, *p* < 0.001, and *p* < 0.0001.

Potential correlations among the five measured adrenal gland dimensions, as well as between sonographic measurements and serum cortisol concentrations, were assessed using Spearman’s rank correlation coefficients.

## 3. Results

### 3.1. General Observations

All ten puppies adapted well to the procedures and exhibited no observable signs of stress during handling at any point throughout the study period. The sonographic evaluation revealed physiologic adrenal gland architecture and echogenicity in all animals, allowing for consistent and reliable measurements. The left adrenal glands appeared bilobed with a narrow central region and two clearly defined poles. In contrast, the right adrenal glands displayed a more comma-shaped morphology, also characterized by a narrow midsection. The adrenal parenchyma was homogeneous in all ten puppies.

### 3.2. Sonographic Parameters

**Figure 1:**
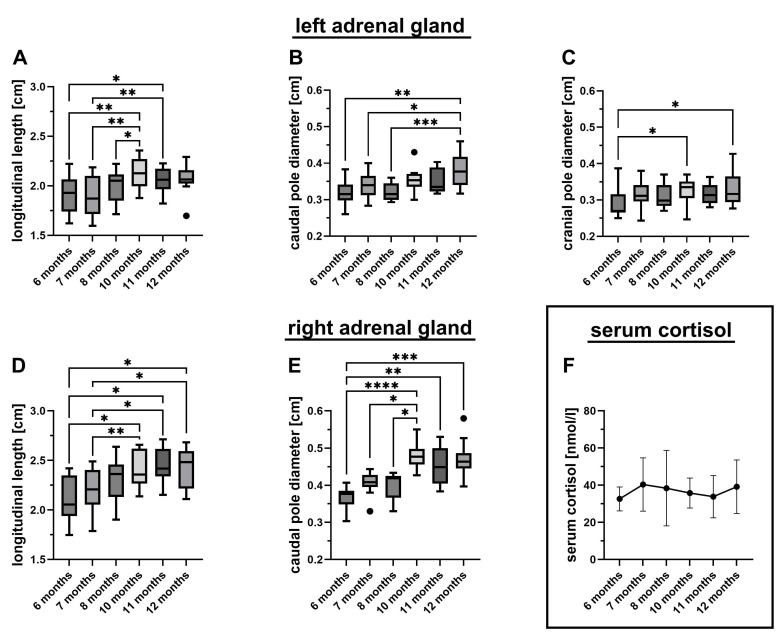
Changes in adrenal gland dimensions and serum cortisol levels over time. The graphs depict the sonographically measured longitudinal length (**A**,**D**), caudal pole diameter (**B**,**E**), and cranial pole diameter (**C**) of the left (**A**–**C**) and right (**D**,**E**) adrenal glands of ten Beagle puppies at different ages, as well as the measured serum cortisol levels (**F**) [20]. Group-wise comparisons revealed significant increases in all adrenal gland dimensions but not in serum cortisol levels (longitudinal length on both sides (**A**,**D**), *p* < 0.0001, one-way repeated-measures ANOVA; the caudal pole diameter on both sides (**B**,**E**), *p* = 0.0001, Friedman test; the left cranial pole diameter (**C**), *p* = 0.0155, Friedman test; and serum cortisol levels (**F**), *p* = 0.6630, one-way repeated-measures ANOVA). Statistical significance of the post hoc tests (Tukey in the case of ANOVA and Dunn’s in the case of the Friedman test) is indicated by * (*p* < 0.05), ** (*p* < 0.01), *** (*p* < 0.001), and **** (*p* < 0.0001).

The adrenal glands of all ten puppies showed a continuous increase in longitudinal length and pole diameter up to ten months of age (Table 1, Figure 1A–E). No statistically significant differences were observed in gland dimensions between ten-, eleven-, and twelve-month-old puppies, suggesting that the adrenal size had reached adult dimensions by ten months. In contrast, the cranial pole of the left adrenal gland appeared to reach its final size earlier, by the seventh month (Figure 1C). The greatest proportional growth was observed in the diameter of the right caudal pole, with an increase of 27.7% (Table 1), while the smallest increase was noted in the longitudinal length of the left adrenal gland, which grew by 8.3%. Except from the longitudinal length of the left adrenal gland in 12-month-old dogs, which was with a mean of 2.15 ± 0.09 cm larger than in males with a mean of 1.97 ± 0.15 cm (*p* < 0.05, Mann Whitney test), we detected no significant sex-specific difference.

### 3.3. Serum Cortisol Levels

Serum cortisol concentrations were predominantly measured within or below the lower end of the reference range, showing considerable variability both within and between individual dogs (Figure 1F). Of the 60 total measurements, 17 values (28%) fell within the laboratory reference range of 41.4 to 96.6 nmol/L. Seven values (12%) were below 22 nmol/L (the basal cortisol cut-off indicative of hypoadrenocorticism, as defined by Gold et al. [20]). Sixty percent of the measurements (36/60) ranged between the lower reference limit and the 22 nmol/L cut-off. On an individual basis, nine out of ten dogs had at least one value within the reference range, and one dog had a single value slightly below (40.6 nmol/L). Seven dogs exhibited one single value below 22 nmol/L. Statistically, the serum cortisol levels did not differ significantly across the six time points (ANOVA), or between males and females at any time point (*t*-tests). The overall mean cortisol concentration was 36.7 ± 13.0 nmol/L, with a median of 34.4 nmol/L and a range of 8.4 to 70.4 nmol/L (Figure 1F).

### 3.4. Correlation Analysis

**Figure 2:**
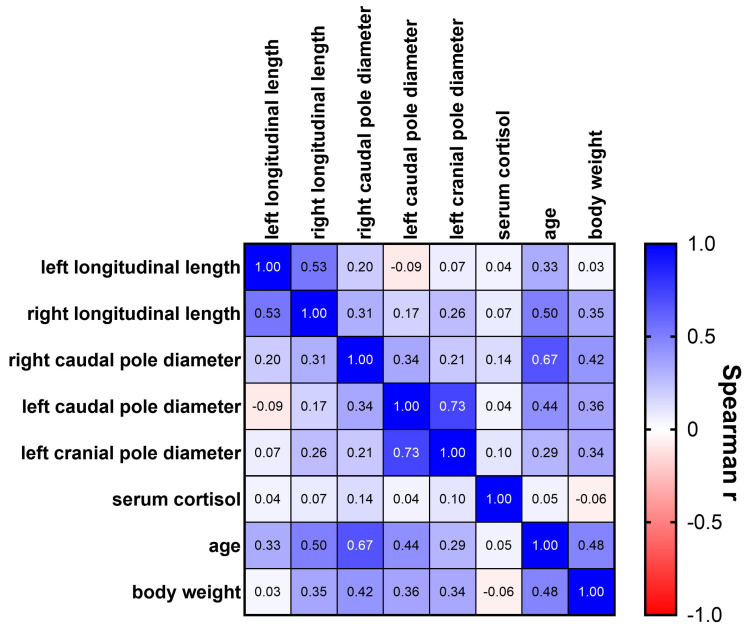
Spearman correlation matrix of measured adrenal gland dimensions, serum cortisol levels, age, and body weight. Spearman correlation coefficients (r) between all assessed parameters are displayed.

Within the measured adrenal gland diameters, moderate correlations, as defined by Chan for medical research [21], were observed between the growth of the left caudal and cranial pole diameters (r = 0.73, *p* < 0.0001; Figure 2), along with fair correlations between the left and right adrenal gland longitudinal lengths (r = 0.53, *p* < 0.0001). Additionally, the diameter of the right caudal pole showed fair correlations with the left caudal pole diameter (r = 0.34, *p* < 0.01) and the right longitudinal length (r = 0.31, *p* < 0.05). The left longitudinal length was not related to any of the pole diameters. No significant associations were found between serum cortisol concentrations and any of the adrenal gland dimensions nor with age or body weight. Age and body weight were correlated with each other (r = 0.48, Figure 2) and, to varying degrees, with all measured adrenal gland dimensions, except for body weight and left longitudinal length.

## 4. Discussion

This study describes the sonographic growth of adrenal glands in Beagle puppies from 6 to 12 months of age, aiming to establish quantitative comparative sizes for the growing adrenal glands. A previous study reported a correlation between adrenal gland size and age in dogs, specifically with the width of the left adrenal gland [13] although no dogs under one year of age were included. In contrast, our findings revealed significant growth in both the longitudinal length and caudal pole diameter, indicating that adrenal gland development continues beyond the initial differentiation into the zona glomerulosa, zona fasciculata, and zona reticularis, typically complete by the third month of life [22]. This suggests a more complex maturation process extending beyond histological development, potentially involving functional adaptations aligned with increasing metabolic and hormonal demands.

Although many studies have established upper reference limits for adrenal gland dimensions to aid in the diagnosis of hyperplasia and neoplasia [7,8,10,14,23], our study focuses on the lower reference range, which is particularly relevant for diagnosing conditions like autoimmune adrenalitis, which is more common in younger dogs [1]. Notably, adrenal glands were visualized in all healthy dogs in previous studies, while in dogs with hypoadrenocorticism, they were sometimes non-visible [1].

The parameters selected in this study were chosen for reproducibility and clinical practicality. The cranial pole, particularly on the right side, is known for high inter- and intraobserver variability, even in computed tomography (CT) studies [24]. In our study, the right cranial pole was excluded due to difficulty in obtaining consistent measurements and signs of patient discomfort. The right adrenal gland is also reported to show higher inter- and intraday variability [10].

Length measurements of the adrenal glands showed the lowest variability across observers, although they are not commonly used in routine practice [10,25]. In this study, both adrenal gland lengths demonstrated clear growth trajectories. Caudal pole thickness, commonly used in clinical sonography due to its reliability, also showed significant growth. Reference ranges for adrenal gland caudal pole thickness vary depending on breed and body size, with values reported between 0.31 and 0.60 cm in the general population [25,26] and 2.2 and 6.6 mm in dogs under 10 kg [25]. In our study, 1.7% of left adrenal and 28% of right adrenal caudal pole measurements fell below 0.32 cm, with 70.3% of all values within the published reference ranges. In order to exclude breed-related specificities in adrenal growth, we focused on Beagle dogs.

Importantly, body weight gain was observed across the study period. In previous findings in dogs <10 kg, no correlation between adrenal gland size and body weight was identified [25]. In the current study, weight correlated with most parameters of the adrenal gland. This might suggest that adrenal growth is not solely determined by physical development but may also be influenced by breed-specific factors or endocrine maturation [27]. While an adrenal gland width <0.39 cm has been associated with an 80% sensitivity and 82% specificity for diagnosing hypoadrenocorticism [28], no single sonographic parameter can definitively exclude the disease.

We observed mild differences in growth dynamics between the left and right adrenal glands, with the right side being consistently larger and exhibiting more uniform growth. The literature on this topic is inconsistent: some studies report larger left adrenal glands, while others show no significant differences, particularly in small breeds [10]. It is possible that the right adrenal gland matures earlier or at a different rate, but further studies involving younger dogs are necessary to confirm this hypothesis. The morphological heterogeneity of adrenal glands—where the right often has a comma- or arrowhead-shape and the left a bilobed or peanut-shaped appearance—may contribute to these discrepancies [10,29], although they appear to lack clinical significance [25].

Basal cortisol levels were generally low or within the lower reference range, consistent with a well-conditioned, minimally stressed population [30,31,32]. Stress significantly impacts cortisol levels; therefore, we implemented a desensitization protocol with positive reinforcement and consistent handlers to minimize stress-induced variability [17,33]. Despite fluctuations, only seven dogs had a single measurement below the 22 nmol/L threshold, which was deemed sufficient to exclude hypoadrenocorticism with high sensitivity and specificity [20]. Additionally, the 28 nmol/L threshold defined by Bovens et al. [34] was rarely breached repeatedly. Although the ACTH stimulation test is considered the gold standard for diagnosing hypoadrenocorticism, it was not conducted in this study. However, none of the dogs exhibited clinical signs of disease during or up to two years after the study, making true hypocortisolism unlikely.

While reference cortisol values typically apply to adult dogs, studies in neonates and puppies with parvoviral enteritis have indicated similar or higher levels [35]. In this study, blood was collected consistently in the morning. According to Palazzo and Quadri (1987), puppies around 8 weeks of age are not fully developed [22,36], which may help explain the observed variability.

Adrenal gland size may also be influenced by sex hormones. In addition to glucocorticoids and mineralocorticoids, the adrenal cortex synthesizes progesterone, estrogens, and androgens [37]. Male Beagles generally reach sexual maturity between 5 and 8 months, and females between 6 and 12 months [38]. The first observed estrus occurred between 8 and 9 months of age. Hormonal changes associated with puberty, as well as social stress resulting from scent detection of sexually mature individuals, may impact adrenal function. Studies suggest that dogs can detect endocrine changes in others, such as stress markers [39,40], potentially influencing adrenal size through social or olfactory cues. Measuring sex hormones parallel to cortisol could provide further insights into these interactions.

This study had several limitations. While the homogeneity of the population (Beagle puppies of similar size and background) improved internal consistency, it limits the generalizability to other breeds. Therefore, the results should be interpreted with care in other breeds. Due to the litter sizes, the study population was quite small, which might reduce statistical power. Nevertheless, a clear growth pattern of the adrenal glands was shown. Additionally, the absence of ACTH stimulation testing prevents definitive conclusions about adrenal function because basal cortisol is influenced by welfare and stress. Further studies will aim to include younger dogs (0–12 months) and, possibly, puppies diagnosed with hypoadrenocorticism. 

## 5. Conclusions

This study evaluated adrenal gland growth in Beagle puppies aged 6 to 12 months using ultrasonography and basal serum cortisol analysis. Over the six-month period, adrenal gland dimensions increased significantly, with growth plateauing around 10 months of age. These findings emphasize the importance of follow-up evaluations of the adrenal glands in growing animals. As most caudal pole measurements in this cohort fell within established adult reference ranges, markedly small adrenal glands should prompt clinicians to consider further functional testing. No correlation was observed between basal cortisol concentrations and adrenal gland size in these healthy Beagle puppies. Therefore, while no single parameter can definitively exclude hypoadrenocorticism, the presence of small adrenal glands in clinically suspicious young dogs should warrant additional diagnostics—such as ACTH stimulation testing—or at a minimum, continued monitoring.

## Figures and Tables

**Table 1:** Assessed sonographic parameters.

	Age [months]	Mean [cm]	Median [cm]	Range [cm]	Growth Rate
Longitudinal length (left)	6	1.92	1.93	1.62–2.22	8.3%
	10–12	2.08	2.08	1.70–2.36	
Longitudinal length (right)	6	2.11	2.06	1.75–2.42	15.1%
	10–12	2.42	2.42	2.11–2.71	
Diameter caudal pole (left)	6	0.32	0.32	0.26–0.38	13.5%
	10–12	0.36	0.36	0.30–0.46	
Diameter caudal pole (right)	6	0.37	0.38	0.30–0.41	27.7%
	10–12	0.47	0.47	0.38–0.58	
Diameter cranial pole (left)	6	0.29	0.27	0.25–0.39	11.7%
	10–12	0.32	0.32	0.25–0.43	

## Data Availability

The raw data supporting the conclusions of this article will be made available by the authors on request.

## Abbreviations

Abbreviation	Meaning
ACTH	Adrenocorticotropic hormone
ANOVA	Analysis of variance
ECLIA	Electrochemiluminescence immunoassay
ECVDI	European College of Veterinary Diagnostic Imaging
kg	Kilogram
MA	Mean average
max.	Maximum
min.	Minimum
mm	Millimeter
nmol/L	Nanomoles per liter
r	Spearman’s correlation coefficient
SD	Standard deviation
e.g.	For example
